# Investigating nurses' perception of bedside handoff

**DOI:** 10.1097/01.NURSE.0000854012.26421.c0

**Published:** 2022-08-25

**Authors:** Adam Winebarger

**Affiliations:** **Adam Winebarger** is the associate chief nursing officer at LifeBridge Health Sinai Hospital in Baltimore, Md.

Bedside shift handoff (BSH) is widely discussed in nursing literature. Its well-established benefits include reducing harmful events and improving communication. Many reasons support the consistent use of BSH as a practice standard in hospitals. Notably, The Joint Commission reports that over 80% of all healthcare errors result from communication breakdowns.^1^

Nurse handoff is defined as “the exchang[e] of vital patient information, responsibility, and accountability between the off-going and oncoming nurses to ensure safe continuity of care and the delivery of best clinical outcomes.”^2^ In contrast to BSH, traditional nursing shift handoff has occurred in a variety of places such as the hallway, nurses' station, or supply closet. Traditional nursing shift handoff is inconsistent, prone to variation, and does not allow clinicians the opportunity to visualize the patient or the care environment at shift change.

BSH encompasses each of the six Domains of Health Care Quality adopted by the Institute of Medicine (now known as The National Academy of Medicine):^3^

- **Safe:** BSH reduces the likelihood of errors and safety events.
- **Effective:** The uniformity of BSH improves staff accountability and the consistency of shared information.
- **Patient-centered:** BSH allows for the personalization and involvement of the patient, family, and caregivers.
- **Timely:** BSH allows for shift-to-shift communication to discuss pertinent matters in real time.
- **Efficient:** BSH provides improved care coordination, improved communication, and mutual understanding of discharge plans.
- **Equitable:** BSH facilitates consistent and improved communication, which is beneficial for patients and families who may be less familiar with hospital processes and terminology.

This article discusses a quality improvement project that explored the nurse perceptions of BSH as a new practice standard in a pediatric hospital. Perceptions of the handoff process were measured before and after the implementation of BSH.

## Identifying a need for change

This project was conducted in a 130-bed pediatric hospital specializing in rehabilitation and transitional care for infants, children, and young adults. The facility had implemented BSH previously, but the efforts were not sustained. After the first implementation in 2014, nurses provided feedback saying that the process took too long or that they feared waking patients and family members from sleep. Some questioned the value of the process. Similar nurse sentiments are also echoed in the literature.^3^,^4^

Routine patient satisfaction surveys indicated a need to improve communication among caregivers. Specifically, patients and family members have commented that nurse communication about care plans seemed disconnected and that each shift was not communicating important events to the next. In addition, leader rounding provided evidence that crucial safety measures, such as ensuring patients were wearing an ID band and scanning the room environment for safety hazards, were missed at shift change. From all of the information gathered, the facility decided to conduct a quality improvement project to implement bedside handoff to improve caregiver communication and reduce the potential for patient safety events.

## Implementation and education

After receiving Institutional Review Board exempt status, all full-time RNs who had been with the organization for longer than 90 days were asked to participate in the *Nursing Assessment of Shift Report*, a 17-item survey that measures perceptions about the handoff process.^5^ The survey measures the perceived efficiency and effectiveness of the handoff process, the likelihood that the handoff process would assist nurses in promptly identifying changes in a patient's condition, nurse stress with handoff, and the extent to which the handoff process promoted teamwork. The instrument is considered valid and reliable.^5^

The first survey was sent to the nurses to get a baseline understanding of their perceptions of the traditional, nonstandardized handoff process. Shortly after, all nursing staff attended a one-time 2-hour interactive training session based on the Agency for Healthcare Research and Quality's BSH implementation toolkit.^6^ Training included didactic instruction, role-play exercises, and a video demonstrating BSH specific to a pediatric setting.^7^ As part of the process and with the input of nursing staff, a new BSH handoff tool was developed using the “ISHAPED” (I=Introduce, S=Story, H=History, A=Assessment, P=Plan, E=Error Prevention, and D=Dialogue) patient-centered handoff approach.^8^ This tool was laminated and placed on all electronic workstations for easy reference during shift changes. The nursing staff also had access to paper copies that they could customize and use for each patient and shift, depending on their preference.

To ensure adherence to the new procedure, nursing leadership staff rounded daily at shift change for 90 days postimplementation. Periodic verification of weekly checks remains ongoing. The most common issue found was staff hesitancy to conduct handoff at the beside when either the patient or the patient's family was sleeping. The staff was encouraged to proceed with the handoff quietly to ensure visualization of patients and their safe environment.

## Results

The survey measuring nurse perceptions was distributed via email to all eligible participants (28) before and after BSH implementation. The pretest survey response rate was 68% (19) and the posttest survey response rate was 75% (21). Responses were gathered after the first survey (see *Shift report question-specific responses*). The result of two independent paired sample t-tests supported the expected outcome that nurses' perceptions of a BSH process would improve significantly after implementing the new process. Aggregate responses were also collected (see *Shift report survey results*).

There was a statistically significant decrease (*P* < .001) in the number of “Strongly Disagree” and “Disagree” responses from the pretest to the posttest surveys, demonstrating improved perceptions. Furthermore, a second paired sample t-test returned a statistically significant increase (*P* < .001) in the number of “Strongly Agree” and “Agree” responses from the pretest to the posttest surveys.

**Table:** Shift report statement-specific responses

	Pre n=19	Post n=21
	Mean Score	Mean Score
The current nursing shift report handover system is an effective means of communication between nurses.	3.43	4.21
The current nursing shift report handover system is an efficient means of communication between nurses.	3.24	4.16
The current nursing shift report handover system help nurses promptly identify changes in patient status.	1.9	4.21
The current nursing shift report handover system helps to ensure nursing accountability.	2.14	4.06
The current nursing shift report handover system helps to ensure that report is given in a professional manner.	2.76	4.12
The current nursing shift report handover system is relatively stress-free.	2.81	4.16
The current nursing shift report handover system provides an opportunity for mentoring/teaching the new nursing staff.	2.2	4
The current nursing shift report handover system promotes patients' involvement in their care.	1.62	4.11
The current nursing shift report handover system helps to prevent delays in patient care and discharge.	1.9	4
The current nursing shift report handover system helps prevent patient safety problems.	2.24	4.06
After receiving the shift/handover report, I feel adequately informed about all aspects of care for my assigned patients.	1.67	4
After receiving the shift/handover report, I feel adequately informed about the plan of care for my assigned patients.	2.1	4.16
After receiving the shift/handover report, I feel adequately informed about the discharge plan for my assigned patients.	2.05	4.21
After receiving the shift/handover report, I feel informed about my assigned patients' need for education/teaching.	2.15	4.16
In general, the shift/handover report is completed within a reasonable amount of time.	2.14	4.16
Nurses on my unit keep patients informed about important aspects of their care (condition, plan of care, options).	1.86	4.16
There is good teamwork between nurses on different shifts on my unit.	1.81	4.11

**Table:** Shift report survey results

Responses	Participants	Score	*P*-value for statistical improvement
Pre-intervention Disagree Responses	19	8.53	*P* ≤ .001∗
Post-intervention Disagree Responses	21	0	
Pre-intervention Agree Responses	19	6.65	P ≤ .001∗
Post-intervention Agree Responses	21	17.47	

∗ indicated statistical evidence in improving the nurses' perception of BSH in this sample group

## Strengths and limitations

One of the project's strengths was the staff's familiarity with BSH, which was not a new concept at the facility. It had been implemented in the past but not sustained. Thus, the learning curve was not significant. The staff and leadership were engaged during the implementation of the project and executive leaders were supportive of the practice change. In informal postimplementation quality audits, the staff reported feeling excited about the process and were satisfied to be recognized for completing the BSH as instructed.

Limitations of the project include the small sample size and response rate. Perception ratings were self-reported. There could have been other factors affecting the responses depending on the patient workload for the day. For example, if the day the survey was completed was particularly stressful, it could be possible the nurse's response was more negative than if the day was going smoothly. The fact that not every eligible RN on the unit participated is also a limitation. The project was conducted in one unit at one facility. The survey did not collect identifiers. No limits were set on the number of surveys submitted from one individual internet protocol address, creating the possibility that respondents may have completed more than one survey at either pre- or postimplementation. Finally, the project was conducted during the COVID-19 pandemic, which potentially affected the responses based on stress, burnout, and other factors.

## Conclusion

Improving communication and reducing errors is a strategic imperative in today's healthcare landscape. The literature supports BSH as a foundational element in a hospital's quest to improve safety and promote better communication among staff, patients, and their family members.^5^,^9^ This quality improvement project's foundational principle was based on improving communication and reducing the potential for harm. Results show statistical evidence of improvement in nurse perceptions of the handoff process after implementation of BSH. This is encouraging and correlates with the facility's strategic plan to improve patient safety.

The expected outcome resulted in a practice change at the facility, from a handoff process performed at the nurse's station to one that now occurs at the bedside. This new process was memorialized in a formal BSH policy implemented in all nursing units at the facility.
